# Brief Report: The Broad Autism Phenotype in Swedish Parents of Children With and Without Autism Spectrum Conditions

**DOI:** 10.1007/s10803-021-05302-3

**Published:** 2021-10-05

**Authors:** Peter Bang, Maria Strömberg, Shoba S. Meera, Kajsa Igelström

**Affiliations:** 1 Division of Neurobiology, Department of Biomedical and Clinical Sciences, University Hospital Campus, Linköping University, 581 85 Linköping, Sweden; 2 Department of Speech Pathology and Audiology, National Institute of Mental Health and Neurosciences (NIMHANS), Bengaluru, India

**Keywords:** Confirmatory factor analysis, Autistic traits, Dimensional measures, Instrument translation

## Abstract

The broad autism phenotype (BAP) is a set of characteristics often observed in typically developing people with a genetic load for autism, such as parents of autistic children. The Broad Autism Phenotypic Questionnaire (BAPQ) is a 36-item questionnaire developed to identify the BAP in first-degree relatives of autistic people. We translated the BAPQ into Swedish and examined its psychometric properties in a Swedish sample consisting of 45 parents of children with ASC and 74 parents of non-autistic children. We found support for the original 3-factor structure (aloof, pragmatic language and rigid), good internal consistency and convergent validity with the Autism Quotient. Thus, the Swedish BAPQ exhibits acceptable psychometric properties and may be useful for assessing the BAP in non-clinical populations.

**Supplementary Information:**

The online version contains supplementary material available at 10.1007/s10803-021-05302-3.

## Introduction

Autism spectrum conditions (ASC) are characterized by social-communicative differences, restricted interests, repetitive behaviors, and sensory abnormalities, which vary across a continuum of severity. ASC has a strong hereditary component, and immediate relatives often show mild to distinct trait-like features in addition to an increased risk for the disorder itself (Bailey et al., 1998). These trait-like features can be understood as a broad autism phenotype (BAP), which refers to a set of subtle but qualitatively similar features to those presented in ASC (Bailey et al., 1998; Bolton et al., 1994). The BAP was first recognized in parents and close relatives of children with ASC (Kanner, 1943) but has since been demonstrated in the general population (Broderick et al., 2015; English et al., 2021; Sasson et al., 2013a, 2013b), supporting the notion of ASC representing an extreme end of a continuous distribution of autism traits. Further support for a genetically based continuum has been found in a meta-analysis of ASC twin studies (Tick et al., 2016), where the combined meta-analytic heritability was estimated to be 64–91%, and in population studies, reporting similarly high heritability of the BAP (62–76%) (Ronald et al., 2005).

Historically, the BAP has been estimated with informant-, interview-, and self-report-based methods such as the Social Responsiveness Scale (SRS) (Constantino et al., 2003), the Family History Interview (FHI) (Bolton et al., 1994) and the Autism Spectrum Quotient (AQ) (Baron-Cohen et al., 2001), respectively. However, common to these instruments is that they were originally designed to measure clinical autistic traits and not the BAP. Hurley et al. specifically developed the BAPQ to capture the BAP features, efficiently and reliably, in non-clinical samples (Hurley et al., 2007). The BAPQ has three subscales with 12 items each; Aloof personality, Rigid personality and Pragmatic language problems, representing the core components of the BAP (Hurley et al., 2007). Studies investigating the psychometric properties of the BAPQ have generally reported good reliability and validity of the BAPQ scores, e.g. showing expected group differences in relatives of autistic probands and controls as well as sex differences (Hurley et al., 2007; Ingersoll et al., 2011; Seidman et al., 2012).

The BAPQ have demonstrated great convergent validity with other often used measures of the BAP (e.g. AQ and SRS) (Ingersoll et al., 2011; Nishiyama et al., 2014; Sasson et al., 2013a, 2013b). Studies of non-clinical populations have also provided evidence of criterion-based validity reporting correlations between BAPQ scores and limited social-cognitive ability (e.g. face processing, emotion recognition and theory of mind) (Sasson et al., 2013a, 2013b). However, some studies have also reported results indicating issues with the factor structure of the BAPQ with unacceptable fit indices (Broderick et al., 2015; Godoy-Giménez et al., 2018) and cross or non-loading items (Godoy-Giménez et al., 2018; Sasson et al., 2013a, 2013b; Stojković et al., 2018; Wainer et al., 2011), especially in the pragmatic language subscale. Despite this, the BAPQ has shown a replicable factor structure supporting its subscales (Ingersoll et al., 2011) and better internal consistency and incremental validity compared to the SRS and the AQ (Ingersoll et al., 2011; Nishiyama et al., 2014). It is easy to administer and does not require clinical expertise. The BAPQ was used for the Simons Simplex Collection (SSC), a vast ongoing repository of genetic samples combined with neuropsychological tests and phenotypic measures from 2600 simplex families (Buxbaum et al., 2014; Nayar et al., 2021). Therefore, it is a valuable dimensional measure for studies of endophenotypes and biological correlates of genetically based autistic traits. The BAPQ has been translated and adapted across different linguistic cultures; however, the instrument does not exist in a Swedish version. We aimed to translate and test the psychometric properties of the BAPQ in a Swedish-speaking population.

## Methods

### The Broad Autism Phenotype Questionnaire, Swedish Version (BAPQ-SE)

We translated the BAPQ into Swedish following recommendations from the World Health Organisation (WHO | Process of translation and adaptation of instruments, n.d.). First, three native Swedish speakers ([names removed]) independently performed forward-translations, and the research group collaboratively resolved any discrepancies. Back-translation was outsourced to a professional English-to-Swedish translator (native English-speaker) who had no prior knowledge of the instrument. The conceptual correspondence of the back-translation and the original was discussed with the original authors, leading to the refinement of some items. Next, we recruited 15 anonymous participants to a cognitive debriefing study administered online. The participants were asked to take the questionnaire and then provide details about any items they found ambiguous or poorly phrased. Comments that pertained to semantic clarity or indicated problems with equivalency were discussed by the team. The main result of the cognitive debriefing was a rephrasing of item 24 (“I act very set in my ways”), from the Swedish equivalent of “I do things in certain specific ways” to “I am inflexible in my ways”, which made more sense to the participants. The final BAPQ-SE can be found in the Supplementary Information.

### Validation Study

The study was exempt from review by the Swedish Ethical Review Authority because no personal data were collected. BAPQ-SE was administered along with the 50-item Autism Quotient (Baron-Cohen et al., 2001) in an online study. The AQ has previously been translated and is widely used in Sweden (Karlsson et al., 2011; Lundin et al., 2019; Lundqvist & Lindner, 2017). Participants were recruited to an online questionnaire (Qualtrics, Seattle, WA) with snowball sampling initiated on social media between mid-December 2020 and mid-January 2021. Recruitment was inclusive and encouraged people with and without autism spectrum conditions to participate. However, we limited the analysis to participants with at least one child for this paper, as this was the population studied originally (Hurley et al., 2007). Exclusion criteria were current psychotic illnesses, brain injury and neurodegenerative disease.

Background questions included age, gender identity, sex assigned at birth, country of birth (Sweden vs other), level of education, parents' level of education, medical conditions, and psychiatric conditions. We also asked how many first-degree relatives the participants had (full siblings, children, parents) and how many of them had a diagnosis of ASC. We did not ask about the children's current age or whether other neurodevelopmental conditions other than ASC were present. For analysis, participants were divided into two groups: parents of children with autism (ASC-parent) and control parents without first-degree relatives with autism (CTRL-parent).

### Statistical Analyses

Analyses were performed using JASP (JASP v.0.14.1, JASP Team, University of Amsterdam, NL). Sex assigned at birth was used as a binary gender variable because no participant reported male-to-female or female-to-male transgender status. The parents' highest educational level was used to approximate socioeconomic status (SES). Education was represented as four levels according to the Swedish school system (corresponding approximately to < 9 years, 9 years, 12 years and > 12 years). Chi-square tests were used to compare sex ratios, education levels and comorbidities between the CTRL-parent and ASC-parent groups.

We performed a multiple regression analysis to assess the influence of demographic variables on the BAPQ score, using ASC-parent, age, sex, education level and SES as independent variables and total BAPQ score as the dependent variable. The data were screened for assumptions and outliers. One outlier (standardized residual > 2.5) was found exerting influence on the regression results. The outlier subject was a male in the ASC-parent group, who showed an extreme response style resulting in a BAPQ score of 1.06. After exclusion of this subject, all assumptions of linearity, normality and homoscedasticity were met, and no multicollinearity was present.

We used Pearson's correlations to test the relationship and convergent validity between AQ and BAPQ subscales. We used Student’s t-tests to compare differences in mean scores between ASC-parent and CTRL-parent. We used a Bonferroni correction to statistically correct for multiple comparisons when needed. The AQ was scored with an ordinal scale (0–3), resulting in a total score range of 0–150. The BAPQ items were scored from 1 to 6 and averaged, resulting in total and subscale scores ranging from 1 to 6 (Hurley et al., 2007).

### Factor Analysis

A confirmatory factor analysis (CFA) was applied to examine the factor structure of the BAPQ-SE. As the items of the BAPQ are measured on an ordinal scale, the diagonally weighted least square (DWLS) procedure was used. This approach leads to less biased parameter estimates, resulting in fewer incorrect standard errors and model test statistics, especially in small sample sizes of N < 150 (Li, 2016; Rhemtulla et al., 2012). The overall goodness of fit was assessed using the Chi-square test statistic, the Standardized Root Mean Square Residual (SRMR) and the Comparative Fit Index (CFI). SRMR signifies the size of the fitted residuals and is a robust measure of model fit across different estimation methods (Shi & Maydeu-Olivares, 2020). SRMR values closer to zero suggest a better fit, and we adopted the cut-off value recommended by Schermelleh-Engel et al. (2003), with < .05 indicating a good fit and < .10 an acceptable fit. The CFI is normed, and values range from zero to one, with higher values indicating a better fit. Schermelleh-Engel et al. (2003) suggest > .97 as indicative of a good fit relative to the independence model, while values > .95 can be interpreted as acceptable.

## Results

### Participant Characteristics

BAPQ and AQ data were collected from 119 parents (81.5% mothers) of autistic children (*N* = 45; ASC-parent) and typically developing children (*N* = 74; CTRL-parent). 93.3% of the cohort had finished high school (≥ 12 years of schooling), and 70.6% reported having completed a university degree. The most common psychiatric conditions present in the parent population were depressive disorders (21.8%), anxiety disorders (13.5%) and attention deficit/hyperactivity disorder (10.1%). No participant reported social communication disorder, and few participants reported obsessive/compulsive disorder (4.2%), apraxia of speech (0.8%) or tic disorder (2.5%). There were no significant group differences in sex ratios, mean age, level of education, SES, or frequency of the psychiatric conditions (Table 1).

**Table 1:** Demographic data

	CTRL-parent	ASC-parent	Test statistic	p value
*N* (females)	74 (57)	45 (40)	χ^2^(1) = 2.613	.106
Age (years ± SD)	53.7 ± 9.8	51.2 ± 9.6	t(117) = 1.345	.181
Education				
Middle school (9 years)	5 (6.8%)	3 (6.7%)		
High school (12 years)	17 (23.0%)	10 (22.2%)	Χ^2^(2) = 0.010	.995
University degree	52 (70.3%)	32 (71.1%)		
Parents’ highest education				
Do not know	5 (6.8%)	2 (4.4%)		
< Middle school	1 (1.4%)	0 (0%)		
Middle school (9 years)	27 (36.5%)	12 (26.6%)	χ^2^(4) = 2.999	.558
High school (12 years)	15 (20.3%)	9 (20.0%)		
University degree	26 (35.2%)	22 (48.9%)		
Psychiatric conditions				
Obsessive/compulsive disorder	2 (2.7%)	3 (6.7%)	χ^2^(1) = 1.092	.296
Speech apraxia	1 (1.4%)	0 (0%)	χ^2^(1) = 0.613	.434
Tic disorders	2 (2.7%)	1 (2.2%)	χ^2^(1) = 0.026	.871
Attention deficit/hyperactivity disorder	6 (8.1%)	6 (13.3%)	χ^2^(1) = 0.843	.359
Depressive disorders	12 (16.2%)	14 (31.1%)	χ^2^(1) = 3.636	.057
Anxiety disorders	9 (12.2%)	7 (15.6%)	χ^2^(1) = 0.277	.599

*ASC-parent* parents of children with autism spectrum conditions, *CTRL-parent* control parents

The multiple regression analysis found a significant regression model, which significantly predicted total BAPQ score (*F*(5,113) = 2.58, *p* = 0.001, adj. *R*^*2*^ = 0.063) (Table S2). Being a parent of a child with ASC was significantly associated with higher BAPQ total scores [*b* = 0.357, *t*(113) = 2.72, *p* = 0.008].

### Descriptive Statistics of the BAPQ

Descriptive statistics of individual BAPQ items are shown in the supplementary material (Table S1). All items showed small to medium values of kurtosis (< 3.0) and skewness (< 1.0) except items 10 and 29 which were extremely skewed (Nasser & Wisenbaker, 2003). The total score of the BAPQ was linearly correlated with total AQ scores (Pearson r = 0.875, p < .001). All BAPQ and AQ subscales were highly intercorrelated (Table 2), except for *Attention to detail* (AQ), which showed weaker correlations with *Rigid personality* (BAPQ) and *Pragmatic language* (BAPQ) and was uncorrelated with *Aloof personality* (BAPQ). The maximum correlations were observed for *Aloof personality* (BAPQ) vs *Social skills* (AQ), *Pragmatic language* (BAPQ) vs *Communication* (AQ), and *Rigid personality* (BAPQ) vs *Attention switch* (AQ) (Table 2). There were significant differences in the BAPQ total scores (p = 0.011, Student's t-test) and the subscale *Aloof personality* scores (p = 0.003) between the two parental groups. This was also true for the AQ total scores and the AQ subscale *Communication* scores (Table 3).

**Table 2:** Relationships between subscale scores of the AQ and BAPQ

	BAPQ-aloof personality	BAPQ-pragmatic language	BAPQ-Rigid personality
AQ-Social skills	0.856*	0.543*	0.587*
AQ-Communication	0.762*	0.775*	0.624*
AQ-Attention switch	0.659*	0.646*	0.831*
AQ-Attention to detail	0.078^ ns^	0.256*	0.375*
AQ-Imagination	0.602*	0.555*	0.442*

Pearson r values, highlighting the top correlation for each BAPQ subscale. Bonferroni corrected p-value cut-off: p < .003 (0.05/15)

*ns* no significance

*Significant after Bonferroni-correction

**Table 3:** Mean BAP measure scores of the two parental groups

	CTRL-parent Mean (SD)	ASC-parent Mean (SD)	t	p-value	Cohen's d
BAPQ					
Rigid personality	2.81 (0.80)	3.00 (0.90)	− 1.195	.235	− 0.226
Aloof personality	2.87 (0.85)	3.37 (0.93)	− 3.004	.003*	− 0.568
Pragmatic language	2.32 (0.53)	2.64 (0.87)	− 2.489	.014	− 0.471
Total score	2.67 (0.62)	3.00 (0.79)	− 2.59	.011*	− 0.49
AQ					
Attention to detail	13.80 (5.76)	13.60 (4.51)	0.196	.845	0.037
Social skills	12.23 (4.98)	14.78 (5.76)	− 2.552	.012	− 0.482
Attention switch	11.37 (4.81)	13.64 (5.89)	− 2.3	.023	− 0.435
Communication	9.05 (4.00)	12.36 (5.60)	− 3.741	< .001*	− 0.707
Imagination	9.26 (4.40)	11.42 (5.05)	− 2.46	.015	− 0.465
Total score	55.70 (17.17)	65.80 (21.66)	− 2.814	.006*	− 0.532

Bonferroni corrected p value cutoff: p < .013 (0.05/4) for BAPQ; p < .008 (0.05/6) for AQ

*ASC-parent* parents of children with autism spectrum conditions, *CTRL-parent* control parents

*Significant after Bonferroni-correction

### Internal Reliability

Inter-item reliability for the full BAPQ scale and each subscale was estimated using the Cronbach's alpha coefficient. Cronbach's alpha was 0.95 for the full 36-item scale, 0.93 for the *Aloof personality* subscale, 0.85 for the *Pragmatic language* subscale, and 0.90 for the *Rigid personality* subscale. Cronbach's alpha was 0.93 for the AQ scale. The three BAPQ subscales were significantly correlated with each other (Pearson’s correlation with Bonferroni-correction for multiple comparisons; *Aloof personality* vs *Rigid personality*, r = 0.63; p < .001, *Aloof personality vs. Pragmatic language*, r = 0.61; p < .001, and *Rigid personality vs. Pragmatic language*, r = 0.60; p < .001; p-value cutoff for α = 0.05 was p = .05/3 = .017).

### Confirmatory Factor Analysis

Bartlett's test of sphericity was statistically significant (χ^2^(630) = 2807.333, p < .001), supporting the use of factor analysis on the dataset. The Kaiser–Meyer–Olkin measure of sampling adequacy was 0.89, indicating strong relationships between the variables. Thus, we performed a CFA on the originally proposed BAPQ three-factor model (Hurley et al., 2007), using robust DWLS estimations. Fit indicators for the BAPQ three-factor model indicated an acceptable fit (χ^2^(591) = 518.495, p = 0.985; SRMR = 0.088; CFI = 1). Factor loadings are shown in Table 4.

**Table 4:** Factor loadings for the BAPQ

Factor	Indicator	Factor loadings			Standardized estimate
		Estimate	95% CI	*P*-value	
Aloof personality	Item 1	0.64	0.49–0.79	< .001	0.65
	Item 5	0.75	0.57–0.93	< .001	0.64
	Item 9	1.01	0.86–1.16	< .001	0.88
	Item 12	0.58	0.37–0.78	< .001	0.52
	Item 16	1.15	0.97–1.33	< .001	0.83
	Item 18	0.82	0.65–0.99	< .001	0.73
	Item 23	1.08	0.89–1.28	< .001	0.72
	Item 25	1.1	0.92–1.28	< .001	0.84
	Item 27	0.74	0.55–0.93	< .001	0.67
	Item 28	0.56	0.38–0.73	< .001	0.57
	Item 31	0.9	0.69–1.10	< .001	0.71
	Item 36	1.2	1.02–1.38	< .001	0.84
Pragmatic language	Item 2	0.85	0.65–1.05	< .001	0.75
	Item 4	0.43	0.16–0.71	< .001	0.33
	Item 7	0.75	0.58–0.92	< .001	0.77
	Item 10	0.43	0.26–0.59	< .001	0.56
	Item 11	1.01	0.80–1.21	< .001	0.85
	Item 14	0.43	0.26–0.61	< .001	0.47
	Item 17	0.48	0.21–0.76	< .001	0.37
	Item 20	0.63	0.32–0.94	< .001	0.5
	Item 21	0.59	0.39–0.79	< .001	0.51
	Item 29	0.36	0.13–0.59	.002	0.35
	Item 32	0.54	0.30–0.79	< .001	0.47
	Item 34	0.91	0.73–1.08	< .001	0.76
Rigid personality	Item 3	0.74	0.50–0.98	< .001	0.6
	Item 6	0.8	0.61–0.99	< .001	0.73
	Item 8	1.0	0.78–1.22	< .001	0.76
	Item 13	0.83	0.58–1.08	< .001	0.64
	Item 15	1.03	0.83–1.24	< .001	0.8
	Item 19	1.06	0.89–1.22	< .001	0.82
	Item 22	0.9	0.69–1.10	< .001	0.74
	Item 24	0.62	0.43–0.82	< .001	0.61
	Item 26	0.63	0.35–0.90	< .001	0.47
	Item 30	0.73	0.53–0.93	< .001	0.6
	Item 33	0.62	0.36–0.87	< .001	0.48
	Item 35	0.57	0.37–0.76	< .001	0.54

## Discussion

Our study demonstrates that the BAPQ-SE is a valid and reliable measure of individual differences in the BAP in a Swedish-speaking population. Having an autistic child was the greatest predictor of high BAPQ scores, whereas demographic variables did not show significant associations with BAPQ scores in this study.

Our population was similar to the parent population used in the original study (Hurley et al., 2007) regarding age, education level and SES, but contained fewer fathers than mothers. The small number of male participants limited statistical power to detect potential sex differences and did not allow us to examine model invariance across sexes. In addition, the majority of participants were highly educated, which might have prevented us from seeing associations with the BAP and education reported previously (Skylark & Baron-Cohen, 2017). The Swedish BAPQ total scores appeared to be higher for the ASC-parent group compared to previous studies, whereas scores for the CTRL-parent group were similar to previous studies (Hurley et al., 2007; Nayar et al., 2021; Sasson et al., 2013a, 2013b; Seidman et al., 2012; Shi et al., 2015); therefore, it may reflect a difference in cohort composition rather than the BAP measure itself. Correlations between the BAPQ and the AQ total scores were also similar to previous studies (Ingersoll et al., 2011; Nishiyama et al., 2014; Sasson et al., 2013a, 2013b; Stojković et al., 2018).

The CFA showed an acceptable fit, with factor loadings above 0.30 for all items, supporting the original three-factor model. Similar to other studies (Godoy-Giménez et al., 2018; Ingersoll et al., 2011; Sasson et al., 2013a, 2013b; Sharma & Bhushan, 2018; Stojković et al., 2018), the *Pragmatic language* subscale had the lowest factor loadings and smallest Cronbach's alpha. The BAPQ-SE had high internal consistency and convergent validity with the AQ, which has been observed in previous studies (Nishiyama et al., 2014; Sasson et al., 2013a, 2013b). Moreover, high BAPQ scores in the ASC-parent group were found for the total score and the *Aloof personality* subscale, but not the other subscales, which is also in line with previous studies (Maxwell et al., 2013; Sasson et al., 2013a, 2013b). These similarities support concurrent validity.

### Limitations

Our recruitment method relied on social media platforms that targeted populations with an active interest in neuropsychiatry. This strategy may have led to the attraction of highly motivated parents and did not allow us to control the representativeness of the sample. The study was entirely anonymous, and we could not assess the BAP clinically as was done in the initial study (Hurley et al., 2007). Previous research with the English BAPQ has demonstrated cut-off scores suitable for identifying the BAP without the need for extensive clinical assessment (Sasson et al., 2013a, 2013b). To determine whether the BAPQ-SE can achieve this purpose reliably and at what cut-off values, clinic-based studies will be needed. The current study indicates that the BAPQ-SE can be used to obtain a dimensional measure of autistic characteristics, e.g. for use in brain imaging studies, but further validation is needed before it can be used to identify BAP in the clinic. The anonymous nature of the study also prevented the use of combined self- and informant-reports (i.e., by spouses), which has been found useful for ameliorating discrepancies between subjective reports and the perspectives of others (Hurley et al., 2007; Sasson et al., 2013a, 2013b; Seidman et al., 2012; Shi et al., 2015).

### Conclusion and Future Directions

Even though the BAPQ was developed for specific use in parents (Hurley et al., 2007; Piven & Sasson, 2014), its high correlation with the AQ indicates that it also gives an approximate measure of subclinical autism traits. Its slightly briefer format and its non-pathologizing language make it an attractive instrument for the quick assessment of autistic traits in neuroscience studies. Having such instruments available in non-English languages gives flexibility to autism researchers and allows broader comparisons between studies internationally. In conclusion, the Swedish version of the BAPQ exhibits acceptable psychometric properties and is useful for assessing the BAP traits in parents of children with ASC. Our study supports the three-factor structure with the subscales *Aloof personality*, *Pragmatic language* and *Rigid personality*.

## Supplementary Information

Below is the link to the electronic supplementary material.

Supplementary file1 (PDF 143 kb)
