# Supplementary material for: Brief Report: The Broad Autism Phenotype in Swedish Parents of Children With and Without Autism Spectrum Conditions
Source: J Autism Dev Disord. 2021 Oct 5;52(10):4575–82. doi: 10.1007/s10803-021-05302-3 (PMC9508042; doi:10.1007/s10803-021-05302-3)
Supplement: Supplementary file 1 — Supplementary file1 (PDF 143 kb) [file 10803_2021_5302_MOESM1_ESM.pdf]

Brief report: The broad autism phenotype in Swedish parents of children with and without autism spectrum conditions

Peter Bang[1], Maria Strömberg[1] & Shoba S. Meera[2], Kajsa Igelström[1]*

[1]Division of Neurobiology, Department of Biomedical and Clinical Sciences, University Hospital Campus, Linköping University, 581 85 Linköping, Sweden

[2]Department of Speech Pathology and Audiology, National Institute of Mental Health and Neurosciences (NIMHANS), Bengaluru, India

*Corresponding author

Kajsa Igelström, kajsa.igelstrom@liu.se

Phone +46 13 281000

Fax +46 13 288909

Contents:

Table S1. Descriptive statistics of BAPQ items 2

Table S2. Multiple regression coefficients 3

BAPQ-SE instructions (Swedish) 3

BAPQ-SE items (Swedish) 4

Supplementary Results

Table S1. Descriptive statistics of BAPQ items

Item number	Mean (SD)	Skewness	Kurtosis
Item 1	3.37 (0.98)	-0.64	-0.25
Item 2	2.70 (1.12)	0.48	0.30
Item 3	3.51 (1.25)	0.28	-0.50
Item 4	3.35 (1.33)	0.37	-0.51
Item 5	2.91 (1.17)	0.34	-0.59
Item 6	2.56 (1.10)	0.51	-0.29
Item 7	2.44 (0.97)	0.55	0.14
Item 8	2.19 (1.31)	0.9	-0.03
Item 9	3.23 (1.15)	-0.09	-0.43
Item 10	1.48 (0.77)	1.78	2.93
Item 11	2.44 (1.18)	0.62	0.15
Item 12	2.43 (1.12)	0.65	0.45
Item 13	2.52 (1.30)	0.58	-0.43
Item 14	2.04 (0.92)	0.59	-0.11
Item 15	2.79 (1.30)	0.52	-0.51
Item 16	3.67 (1.38)	-0.14	-0.68
Item 17	2.49 (1.29)	0.74	0.06
Item 18	2.65 (1.13)	0.2	-0.52
Item 19	3.12 (1.30)	-0.01	-0.87
Item 20	2.31 (1.27)	0.97	0.57
Item 21	2.51 (1.16)	0.2	-1.09
Item 22	2.70 (1.22)	0.63	0.12
Item 23	3.44 (1.51)	0.02	-0.81
Item 24	2.34 (1.01)	0.93	0.89
Item 25	3.00 (1.30)	0.16	-0.63
Item 26	2.63 (1.34)	0.79	0.17
Item 27	2.76 (1.10)	0.38	0.47
Item 28	2.25 (0.98)	0.47	-0.24
Item 29	1.97 (1.03)	1.08	1.28
Item 30	4.24 (1.23)	-0.7	0.48
Item 31	3.35 (1.26)	0.31	-0.15
Item 32	2.76 (1.15)	0.66	0.30
Item 33	3.35 (1.29)	0.42	-0.37
Item 34	2.82 (1.20)	0.16	-0.70
Item 35	2.61 (1.04)	0.51	0.17
Item 36	3.62 (1.43)	-0.29	-0.71

Abbreviations: SD, standard deviation

Table S2. Multiple regression coefficients

							Collinearity Statistics	
**Model**		**Unstandardized**	**Standard Error**	**Standardized**	**t**	**p**	**Tolerance**	**VIF**
1	(Intercept)	3.391	0.655		5.179	< .001		
	ASC-parent (1/0)	0.357	0.131	0.248	2.719	0.008	0.958	1.044
	Age (years)	-0.008	0.007	-0.118	-1.236	0.219	0.873	1.145
	Male sex (1/0)	0.256	0.167	0.142	1.537	0.127	0.929	1.076
	Education level (1, 2, 3, 4)	-0.137	0.106	-0.118	-1.284	0.202	0.94	1.064
	SES (1, 2, 3, 4)	-0.032	0.061	-0.052	-0.524	0.602	0.814	1.229

Abbreviations: VIF, variance inflation factor; ASC-parent, parents of children with autism spectrum conditions; SES, socioeconomic status.

BAPQ-SE

Enkät om Personlighetstyp och Preferenser

Instruktioner

Du kommer strax att få svara på en rad påståenden som rör personlighet och livsstil. På varje fråga ber vi dig ringa in det svar som bäst beskriver hur ofta det påståendet stämmer in på dig.

Många av frågorna rör ditt samspel med andra människor. **Tänk på hur du är med de flesta människor snarare än speciella relationer** som man kan ha med makar eller partner, barn, syskon och föräldrar.

Alla förändras med tiden, vilket kan göra det svårt att svara på frågor om personlighet. **Tänk på hur du har varit under den största delen av ditt vuxna liv** snarare än hur du var som tonåring eller tillfällen när du känt dig annorlunda än vanligt.

Du måste svara på alla frågor och får bara ge ett svar per fråga. Om du är osäker ber vi dig ange din bästa gissning.

[Likertskala: Väldigt sällan (1), Sällan (2), Ibland (3), Rätt ofta (4), Ofta (5), Väldigt ofta (6)]

1. Jag gillar att umgås med andra människor.
2. Jag har svårt att få ur mig det jag vill säga på ett smidigt sätt.
3. Jag känner mig bekväm med oväntade ändringar av mina planer.
4. Det är svårt för mig att undvika att komma in på sidospår under samtal.
5. Jag skulle hellre prata med människor för att få information än för att umgås.
6. Andra måste övertala mig att prova något nytt.
7. Jag är engagerad och lyhörd när jag pratar med andra.
8. Jag måste vänja mig vid tanken på att besöka en främmande plats.
9. Jag trivs i sociala situationer.
10. Mitt röstläge är monotont och saknar melodi.
11. Jag känner mig avskärmad eller ”ur fas” i samtal med andra.
12. Andra människor tycker att det är lätt att konversera med mig.
13. Jag känner ett stort behov av att mina dagliga rutiner ser likadana ut.
14. Andra ber mig upprepa vad jag sagt för att de inte förstår vad jag säger.
15. Jag är flexibel när det gäller hur saker och ting ska göras.
16. Jag ser fram emot situationer där jag kan träffa nya människor.
17. Jag har fått höra att jag pratar för mycket om vissa ämnen.
18. När jag samtalar är det bara för att vara artig.
19. Jag tycker om att prova nya saker.
20. Jag pratar med för stark eller för svag röst.
21. Jag märker när någon inte är intresserad av vad jag säger.
22. Jag tycker det är jobbigt när mina rutiner förändras.
23. Jag är bra på att småprata.
24. Jag är oflexibel i mitt sätt att vara.
25. Det känns som om jag verkligen får bra kontakt med andra människor.
26. Andra blir frustrerade över min ovilja att ge mig.
27. Samtal tråkar ut mig.
28. Jag är varm och vänlig i mitt samspel med andra.
29. När jag samtalar gör jag långa uppehåll innan jag forsätter att prata.
30. Jag varierar mina dagliga rutiner genom att prova att göra dem på nya sätt.
31. Jag är hellre ensam än tillsammans med andra.
32. När jag pratar med andra glider jag lätt bort ifrån samtalsämnet.
33. Jag gillar att följa en rutin noga när jag arbetar.
34. Jag märker när det är dags att byta ämne i ett samtal.
35. Jag fortsätter att göra saker på det sätt jag känner till, även om ett annat sätt kan vara bättre.
36. Jag tycker om att småprata med människor.
